# General practice chest X-ray rate is associated with earlier lung cancer diagnosis and reduced all-cause mortality: a retrospective observational study

**DOI:** 10.3399/BJGP.2024.0466

**Published:** 2025-03-25

**Authors:** Stephen H Bradley, Richard D Neal, Matthew EJ Callister, Benjamin Cornwell, William T Hamilton, Gary A Abel, Bethany Shinkins, Richard B Hubbard, Matthew E Barclay

**Affiliations:** School of Medicine and Population Health, University of Sheffield, Sheffield.; Department of Health and Community Sciences, University of Exeter, Exeter.; Department of Respiratory Medicine, Leeds Teaching Hospitals; Leeds Institute of Health Sciences, University of Leeds, Leeds.; Royal United Hospitals, Bath.; Department of Health and Community Sciences, University of Exeter, Exeter.; Department of Health and Community Sciences, University of Exeter, Exeter.; Warwick Applied Health, Warwick Medical School, Coventry.; School of Medicine, University of Nottingham, Nottingham.; Epidemiology of Cancer Healthcare and Outcomes, Department of Behavioural Science and Health, Institute of Epidemiology and Health Care, University College London, London.

**Keywords:** chest X-ray, general practice, lung cancer, early detection of cancer

## Abstract

**Background:**

Evidence is equivocal on whether general practice rates of investigation in symptomatic patients using chest X-ray (CXR) affect outcomes.

**Aim:**

To determine whether there is an association between rates of CXR requested in general practice and lung cancer outcomes.

**Design and setting:**

Observational study using data on English general practices.

**Method:**

Cancer registry data for patients diagnosed with lung cancer in 2014–2018 were linked to data on general practice CXRs from 2013 until 2017. Cancer stage at diagnosis (I/II versus III/IV) and 1-year and 5-year survival rates (conditional on survival to 1 year) post-diagnosis were reported by general practice quintile of CXR rate, with adjustment for population differences (age, smoking, prevalence of chronic obstructive pulmonary disease and heart failure, ethnicity, and deprivation) and by unadjusted category (low, medium, and high).

**Results:**

In total, 192 631 patient records and CXR rates for 7409 practices were obtained. Practices in the highest quintile of CXR rate had fewer cancers diagnosed at stage III/IV compared with those in the lowest quintile (odds ratio [OR] 0.87, 95% confidence interval [CI] = 0.83 to 0.92, *P*<0.001). The association was weaker for the high unadjusted CXR category (OR 0.94, 95% CI = 0.91 to 0.97). For the highest adjusted quintile, hazard ratios (HRs) for death within 1 year and 5 years were 0.92 (95% CI = 0.90 to 0.95, *P*<0.001) and 0.95 (95% CI = 0.91 to 0.99, *P* = 0.023), respectively. For the high unadjusted CXR category, the HR for 1-year survival was 0.98 (95% CI = 0.96 to 0.99, *P* = 0.004), with no association demonstrated for 5-year survival.

**Conclusion:**

Patients registered at general practices with higher CXR use have a favourable stage distribution and slightly better survival. This supports the use of CXR in promoting earlier diagnosis of symptomatic lung cancer in general practice.

## Introduction

Curative treatment of lung cancer depends largely on diagnosis before progression to advanced disease; survival is closely associated with less-advanced stage of disease at diagnosis.^1^ The importance of lung cancer detection in asymptomatic individuals is widely recognised, with approval for risk -stratified screening from the relevant authorities in countries including the US and the UK.^2^^,^^3^ However, only half of patients diagnosed with lung cancer would have been eligible for screening,^4^ and barely more than half of those who are eligible accept their invitation.^5^^,^^6^ Even in the context of effective national screening programmes, improving timely diagnosis in those with symptoms remains crucial.

Clinical guidelines worldwide recommend chest X-ray (CXR) as the initial investigation for common symptoms of lung cancer, such as cough and shortness of breath, with few exceptions.^7^ Recommendations for referral to specialists and/or investigation using computed tomography (CT) are commonly reserved for presentations conferring a higher risk of lung cancer, such as haemoptysis, although this symptom of the disease occurs relatively infrequently.^7^^,^^8^ CXR has much lower sensitivity than CT,^9^ but its widespread availability at relatively low cost supports its role as a first-line investigation for common symptoms, particularly in healthcare systems in which CT availability is limited.^10^

Volumes of diagnostic activity, including suspected lung cancer referrals and CXRs, vary substantially between primary healthcare services, even after adjustment for population differences and the role of chance.^11^^,^^12^ Previous research has shown that patients attending general practices that undertake more urgent referrals for suspected lung cancer have the disease detected at earlier stages and have improved survival.^13^ Many, or perhaps most, of such referrals would have been prompted by abnormal findings on CXR,^14^ but it is not known whether general practices that utilise CXR more frequently are more likely to detect lung cancer at earlier stages and whether patients have improved survival as a result. To ascertain this, the authors examined routinely collected national data for England.

**Table:** How this fits in

It is known that there is wide variation in the use of chest X-ray (CXR) by general practices, but previous studies have provided conflicting evidence as to whether greater utilisation of them leads to lung cancer being diagnosed at an earlier stage and improves survival. This observational study analysed data from the English national cancer registry on CXR rates for individual general practices, along with stage and survival outcomes; it found earlier stage at diagnosis and improved survival for patients diagnosed with cancer at practices that used the test more frequently. Increasing use of CXR by GPs for symptomatic patients, particularly by focusing on practices that use the test infrequently, could improve lung cancer outcomes.

## Method

### Data sources

#### General practice CXR rates and population characteristics

The authors obtained annual counts of CXRs (rounded to the nearest five) requested by English general practices with ≥1000 registered patients in England from the NHS’s Diagnostic Imaging Dataset (DID)^15^ for 2013–2017. On the advice of DID staff, practice CXR total counts of <5 for any single year were excluded as such low volumes were likely to be erroneous.

Additional data on the general practices were obtained from published general practice profiles,^16^ permitting adjustment for practice list size and characteristics of patient populations, such as age, sex, ethnicity, Index of Multiple Deprivation (IMD) quintile, prevalence of smoking, and prevalence of specific comorbidities associated with variation in CXR rates (namely, heart failure and chronic obstructive pulmonary disease).^12^

General practices were categorised by CXR counts in two ways. Mixed-effects Poisson modelling was used to adjust for the characteristics listed above and for chance variation, with practices ranked into quintiles by adjusted CXR use, using the methodology previously described.^12^ In addition, practices were allocated to one of three categories, specified with reference to the median CXR rate determined in previous work to be 34 CXRs per 1000 patients (interquartile range 26–43),^12^ without adjustment for practice population characteristics. These three categories were:

- low — <30 CXRs per 1000 patients;
- typical — 30–39 CXRs per 1000 patients; and
- high — ≥40 CXRs per 1000 patients.

The rationale for creating these unadjusted categories was to provide categories for health service managers and policymakers that could be easily interpreted.

For both the adjusted and unadjusted sets of categories, composite values were created to represent the entire period of 2013–2017. These composite values, rather than values for single years, were used for the primary outcomes. Composite values for the adjusted and unadjusted CXR rates were only created if there were valid data for at least three of the years in the period 2013–2017. For the adjusted CXR rate, the best linear unbiased prediction (BLUP) of the practice-level effect was used to assign a CXR rate category. For the unadjusted rate, practices were categorised based on the BLUP applied to the mean CXR rate in 2017.

Adjusted and unadjusted categorisation of practices, including for individual years from 2013 until 2017 and for the entire 5-year period, along with data on practice characteristics used in mixed-effects Poisson regression to support adjustment are available online (https://osf.io/k2tm5).

#### Lung cancer outcomes data

The authors obtained data from the National Cancer Registration and Analysis Service (NCRAS) on patients who were diagnosed with primary lung cancer in England from 2014 until 2018. Specifically, lung cancer stage at diagnosis (I, II, III, and IV, recorded in either the seventh or eighth edition of the tumour, node and metastasis [TNM] classification system, depending on the date) and survival in days following the date of diagnosis were extracted, along with the following covariables: age (5-year bands, other than 18–24 years and ≥100 years), sex (male/female), ethnicity (White, Mixed, Asian, Black, Chinese, and Other), Charlson Comorbidity Index score (0, 1, and ≥2), and IMD quintile. The adjusted and unadjusted general practice categories described above were assigned to patients according to the practices at which they were registered at the time of diagnosis. For patients who had >1 primary lung cancer diagnosed during 2014–2018, only the first cancer diagnosed in that period was considered.

#### Data for exploratory analyses

In addition to data on general practices’ CXR rates, the authors also obtained data, including general practice-requested CXRs, at individual level (NCRAS-DID*-*linked data). Further detail on data obtained for exploratory analyses are available in the analysis plan.^17^

### Statistical analysis

Statistical analysis for primary outcomes, sensitivity analyses, and exploratory analyses were undertaken in accordance with a pre-registered analysis plan.^17^ The authors examined associations with stage at diagnosis by estimating odds ratios (ORs) for diagnosis at advanced stage (III or IV) versus non-advanced stage (I or II) with respect to practice CXR category using logistic regression with stage as the outcome variable, the patient-level covariates described above, and the general practice CXR rate category. Patients with missing stage information were excluded.

Initially, Kaplan–Meier curves were used to describe the risk of death (all causes) post-diagnosis of lung cancer for each of the practice CXR rate categories. Cox proportional hazards regression was then used to estimate the hazard ratio (HR) for death (all causes) after diagnosis of lung cancer, with respect to the practice CXR rate categories. The survival models did not adjust for stage at diagnosis, as this was likely to be the primary route by which differences in CXR use could affect lung cancer survival. HRs were examined for death in two periods: from diagnosis to 1 year post-diagnosis, and from 1 year post-diagnosis to 5 years post-diagnosis.

All logistic and Cox proportional hazard regressions used cluster-robust standard errors to account for possible clustering in outcomes for patients registered at the same general practice. Statistical analyses were undertaken using Stata (version 17.0).

### Sensitivity and exploratory analyses

Sensitivity analyses considered:

- exclusion of patients aged <40 years at date of diagnosis to reflect imaging guidance from the National Institute for Health and Care Excellence;^18^
- using age as a continuous variable; and
- using CXR categories from the year before diagnosis (rather than the entire period of 2013–2017).

Further exploratory analyses included examination of whether:

- season affected CXR rate and stage of diagnosis; and
- individuals who were investigated more frequently with CXR in the years prior to diagnosis (excluding the first year) were diagnosed at earlier stages of the disease.

## Results

**Figure 1.**
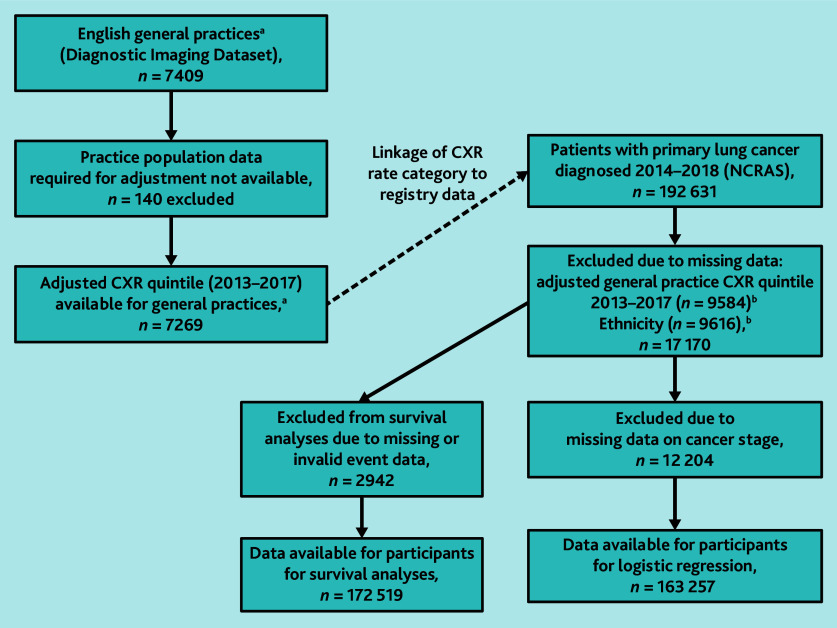
Population for primary analyses using adjusted practice CXR rate. ^a^General practices with ≥1000 patients for which ≥3 CXRs were performed for at least 3 individual years in 2013–2017. ^b^Total is not 17 170 as both variables are missing for several participants. CXR = chest X-ray. NCRAS = National Cancer Registration and Analysis Service.

**Figure 2.**
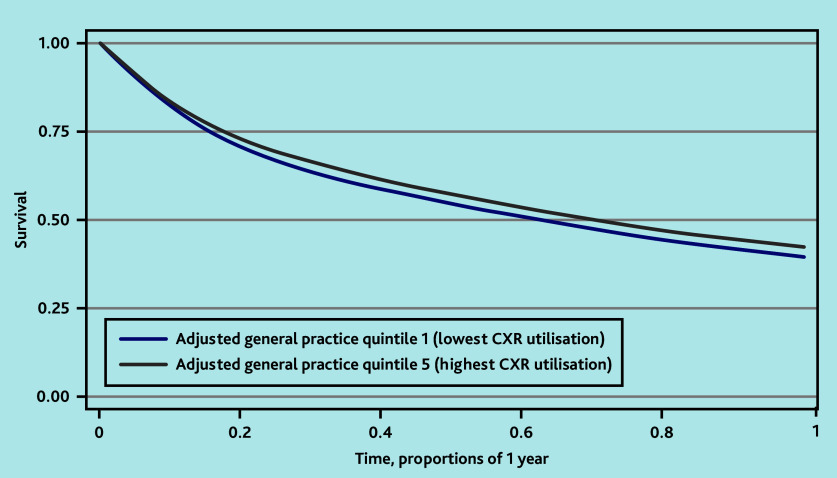
One-year Kaplan–Meier survival curves for adjusted practice CXR quintiles 1 versus 5. ^a^*n* = 172 519 observations; *n* = 102 753 censored by year 1. CXR = chest X-ray.

Data on 192 631 individuals diagnosed with lung cancer in the period 2014–2018 were obtained from NCRAS, and CXR rates for the period 2013–2017 were obtained for 7409 general practices (Figure 1 and Supplementary Figure S1). After excluding cases with missing data, analyses of cancer stage at diagnosis comprised 163 257 participants and 165 240 participants using adjusted and unadjusted CXR categories, respectively, while survival analyses comprised 172 519 participants (Figures 1 and 2) and 173 789 participants. Table 1 summarises the characteristics of participants in these analyses, and Supplementary Table S1 summarises data for those who were excluded.

**Table 1.** Characteristics of participants included in primary analyses

**Characteristic**	**Logistic regression using adjusted CXR quintile**	**Survival analysis using adjusted CXR quintile**	**Logistic regression using unadjusted CXR rate**	**Survival analysis using unadjusted CXR rate**
**Total, *n***	163 257	172 519	165 240	173 789

**Sex, male, *n* (%)**	86 461 (53.0)	91 125 (52.8)	87 524 (53.0)	91 781 (52.8)

**Mean age, years (SD)**	72 (10.6)	72 (10.7)	72 (10.6)	72 (10.7)

**Ethnicity, *n* (%)^a^**				
White	156 716 (96.0)	165 591 (96.0)	158 556 (96.0)	166 813 (96.0)
Mixed	465 (0.3)	489 (0.3)	476 (0.3)	493 (0.3)
Asian	2458 (1.5)	2619 (1.5)	2504 (1.5)	2634 (1.5)
Black	1727 (1.1)	1813 (1.1)	1764 (1.1)	1829 (1.1)
Chinese	369 (0.2)	393 (0.2)	378 (0.2)	399 (0.2)
Other	1522 (0.9)	1614 (0.9)	1562 (0.9)	1621 (0.9)

**IMD quintile, *n* (%)**				
1 (least deprivation)	23 184 (14.2)	24 469 (14.2)	23 493 (14.2)	24 678 (14.2)
2	29 241 (17.9)	30 900 (17.9)	29 586 (17.9)	31 118 (17.9)
3	32 400 (19.8)	34 243 (19.8)	32 767 (19.8)	34 471 (19.8)
4	35 636 (21.8)	37 737 (21.9)	36 074 (21.8)	38 014 (21.9)
5 (most deprivation)	42 796 (26.2)	45 170 (26.2)	43 319 (26.2)	45 508 (26.2)

**Charlson Comorbidity Index score, *n* (%)**				
0	109 138 (66.9)	115 296 (66.8)	110 460 (66.8)	116 151 (66.8)
1	23 045 (14.1)	24 337 (14.1)	23 316 (14.1)	24 516 (14.1)
≥2	31 074 (19.0)	32 886 (19.1)	31 464 (19.0)	33 122 (19.1)

**Stage at diagnosis, *n* (%)**				
I	31 623 (19.4)	31 563 (18.3)	32 009 (19.4)	31 779 (18.3)
II	13 402 (8.2)	13 380 (7.8)	13 540 (8.2)	13 470 (7.8)
III	34 572 (21.2)	34 495 (20.0)	34 932 (21.1)	34 745 (20.0)
IV	83 660 (51.2)	83 385 (48.3)	84 759 (51.3)	84 014 (48.3)
Unknown		9696 (5.6)		9781 (5.6)

**Histology, *n* (%)**				
Small cell carcinoma	16 993 (10.4)	17 435 (10.1)	17 197 (10.4)	17 570 (10.1)
Adenocarcinoma	53 976 (33.1)	55 363 (32.1)	54 557 (33.0)	55 747 (32.1)
Squamous cell carcinoma	31 257 (19.1)	31 923 (18.5)	31 562 (19.1)	32 147 (18.5)
Other non-small cell carcinoma	23 780 (14.6)	25 501 (14.8)	24 116 (14.6)	25 706 (14.8)
Other	4457 (2.7)	4995 (2.9)	4504 (2.7)	5030 (2.9)
Unknown	32 794 (20.1)	37 302 (21.6)	33 304 (20.2)	37 589 (21.6)

a *The cancer registry uses ethnicity categories based on the 2001 census. For this study, categories were grouped as follows: White = (White) British, (White) Irish, and any other White background; Mixed = White and Black Caribbean, White and Black African, White and Asian, and any other mixed background; Asian = Indian, Pakistani, Bangladeshi, and any other Asian background; Black = Caribbean, African, and any other Black background; Chinese = Chinese; and Other = any other ethnic group. CXR = chest X-ray. IMD = Index of Multiple Deprivation. SD = standard deviation.*

### Association between practice CXR rate and stage at diagnosis

Patients registered at practices in the highest adjusted quintile of CXR utilisation had the lowest odds of being diagnosed at advanced stage (stage III or IV) (OR 0.87, 95% confidence interval [CI] = 0.83 to 0.92, *P*<0.001 compared with the lowest adjusted quintile of CXR utilisation). This was followed by patients registered at practices in the second highest quintile (OR 0.93, 95% CI = 0.89 to 0.98, *P* = 0.004) (Table 2).

**Table 2.** Logistic regression reporting ORs of late-stage (stage III/IV) compared with early-stage (stage I/II) cancer at diagnosis

**Characteristic**	**OR**	***P*-value**	**95% CI**
**Adjusted CXR quintile**			
1 (least use)^a^	1.000		
2	1.017	0.511	0.968 to 1.068
3	0.993	0.781	0.947 to 1.042
4	0.932	0.004	0.888 to 0.978
5 (highest use)	0.874	<0.001	0.828 to 0.922

**Sex**			
Male^a^	1.000		
Female	0.781	<0.001	0.764 to 0.798

**Age group, years**			
18–24	0.166	<0.001	0.086 to 0.322
25–29	0.367	<0.001	0.241 to 0.558
30–34	0.592	0.001	0.432 to 0.812
35–39	1.058	0.699	0.826 to 1.329
40–44	1.473	<0.001	1.246 to 1.742
45–49	1.568	<0.001	1.416 to 1.737
50–54	1.403	<0.001	1.306 to 1.508
55–59	1.212	<0.001	1.147 to 1.280
60–64	1.068	0.004	1.021 to 1.116
65–69^a^	1.000		
70–74	0.905	<0.001	0.873 to 0.939
75–79	0.853	<0.001	0.822 to 0.885
80–84	0.876	<0.001	0.842 to 0.912
85–89	0.951	0.033	0.908 to 0.996
90–94	1.137	<0.001	1.058 to 1.222
95–99	1.293	0.004	1.087 to 1.537
≥100	2.487	0.059	0.965 to 6.408

**Ethnicity**			
White^a^	1.000		
Mixed	1.227	0.066	0.986 to 1.527
Asian	0.955	0.342	0.870 to 1.050
Black	1.010	0.854	0.906 to 1.126
Chinese	0.894	0.317	0.717 to 1.114
Other	1.155	0.019	1.024 to 1.302

**Charlson Comorbidity Index score**			
0^a^	1.000		
1	0.985	0.346	0.954 to 1.017
≥2	1.017	0.258	0.988 to 1.046

**IMD quintile**			
1 (least deprivation)^a^	1.000		
2	0.961	0.044	0.925 to 0.999
3	0.961	0.035	0.925 to 0.997
4	0.990	0.602	0.954 to 1.028
5 (most deprivation)	1.004	0.831	0.969 to 1.040

a *Reference category. CXR = chest X-ray. IMD = Index of Multiple Deprivation. OR = odds ratio.*

Similarly, patients registered at practices categorised in the high unadjusted general practice CXR rate category (≥40 CXRs/1000 patients) were less likely to be diagnosed at late stage than patients at practices in the low category (OR 0.94, 95% CI = 0.91 to 0.97, *P*<0.001) (see Supplementary Table S2). The full results of the logistic regression with respect to cancer-stage category for adjusted CXR quintiles is detailed in Table 2; Supplementary Table S2 details the findings for unadjusted CXR categories.

Other factors associated with earlier-stage diagnosis were female sex (OR 0.78, 95% CI = 0.76 to 0.80) and the following age categories: 30–34 years and younger, and 70–74 years up to and including 80–84 years. No association was demonstrated between Charlson Comorbidity Index score or ethnicity and early-stage diagnosis. There was weak evidence of an association between deprivation and early diagnosis (Table 2), with ORs for IMD quintiles 2 and 3 being 0.96 (95% CI = 0.93 to 1.00, *P* = 0.044) and 0.96 (95% CI = 0.93 to 1.00, *P* = 0.035) compared with those for IMD quintile 1 (least deprived).

### Association between practice CXR rate and survival

Patients registered at practices in the adjusted CXR quintile with the highest utilisation — namely, quintile 5 — had better survival at 1 year than patients at practices in the lowest quintile (Figure 2), with further advantages seen at 5 years (see Supplementary Figure S2). This was reflected in the HRs obtained from the Cox regression for death within 1 year post-diagnosis (HR 0.92, 95% CI = 0.90 to 0.95, *P*<0.001) (Table 3) and within 5 years (HR 0.95, 95% CI = 0.91 to 0.99, *P* = 0.023) (Table 4). Cox regression 5-year survival data including all patients (not just those who survived to at least 1 year) are reported in Supplementary Table S3.

**Table 3.** Results of Cox regression survival analysis for survival 1 year after date of diagnosis

**Characteristic**	**HR^a^**	***P*-value**	**95% CI**
**Adjusted CXR quintile**			
1 (least use)	1.000		
2	1.005	0.678	0.980 to 1.031
3	0.979	0.095	0.955 to 1.004
4	0.956	<0.001	0.932 to 0.980
5 (highest use)	0.923	<0.001	0.898 to 0.949

**Sex**			
Female	0.814	<0.001	0.804 to 0.824
Male	1.000		

**Age group, years**			
18–24	0.158	<0.001	0.072 to 0.345
25–29	0.304	<0.001	0.200 to 0.462
30–34	0.509	<0.001	0.394 to 0.659
35–39	0.639	<0.001	0.547 to 0.748
40–44	0.827	<0.001	0.756 to 0.905
45–49	0.892	<0.001	0.845 to 0.942
50–54	0.907	<0.001	0.872 to 0.944
55–59	0.917	<0.001	0.889 to 0.946
60–64	0.942	<0.001	0.918 to 0.966
65–69	1.000		
70–74	1.064	<0.001	1.041 to 1.087
75–79	1.204	<0.001	1.178 to 1.230
80–84	1.413	<0.001	1.382 to 1.446
85–89	1.704	<0.001	1.661 to 1.747
90–94	2.185	<0.001	2.112 to 2.260
95–99	2.689	<0.001	2.498 to 2.895
≥100	3.447	<0.001	2.586 to 4.594

**Ethnicity**			
White	1.000		
Mixed	0.891	0.049	0.795 to 1.000
Asian	0.779	<0.001	0.739 to 0.821
Black	0.840	<0.001	0.789 to 0.894
Chinese	0.565	<0.001	0.483 to 0.660
Other	0.899	0.001	0.843 to 0.959

**Charlson Comorbidity Index score**			
0	1.000		
1	0.995	0.595	0.977 to 1.013
≥2	1.000	0.997	0.984 to 1.016

**IMD quintile**			
1 (least deprivation)	1.000		
2	0.962	<0.001	0.941 to 0.982
3	0.967	0.002	0.947 to 0.988
4	0.982	0.077	0.962 to 1.002
5 (most deprivation)	0.979	0.035	0.960 to 0.998

a *HRs reported with respect to death within 1 year after date of diagnosis, first for adjusted general practice CXR quintile, then covariables. CXR = chest X-ray. HR = hazard ratio. IMD = Index of Multiple Deprivation.*

**Table 4.** Cox regression survival analysis for 5-year survival (including only those who survived at least to 1 year, *n* = 66 641 observations) after date of diagnosis

**Characteristic**	**HR**	***P*-value**	**95% CI**
**Adjusted CXR quintile**			
1 (least use)	1.000		
2	0.994	0.756	0.957 to 1.032
3	0.978	0.249	0.943 to 1.015
4	0.965	0.064	0.930 to 1.002
5 (highest use)	0.953	0.023	0.914 to 0.993

**Sex**			
Female	0.792	<0.001	0.777 to 0.808
Male	1.000		

**Age group, years**			
18–24	0.113	<0.001	0.042 to 0.309
25–29	0.197	<0.001	0.107 to 0.362
30–34	0.459	<0.001	0.331 to 0.638
35–39	0.588	<0.001	0.477 to 0.724
40–44	0.725	<0.001	0.627 to 0.838
45–49	0.858	<0.001	0.787 to 0.935
50–54	0.927	0.016	0.872 to 0.986
55–59	0.901	<0.001	0.859 to 0.946
60–64	0.940	0.002	0.903 to 0.978
65–69	1.000		
70–74	1.085	<0.001	1.050 to 1.122
75–79	1.236	<0.001	1.196 to 1.278
80–84	1.463	<0.001	1.412 to 1.515
85–89	1.837	<0.001	1.765 to 1.911
90–94	2.345	<0.001	2.204 to 2.496
95–99	2.843	<0.001	2.471 to 3.271
≥100	2.089	0.003	1.284 to 3.397

**Ethnicity**			
White	1.000		
Mixed	1.024	0.792	0.860 to 1.218
Asian	0.879	0.001	0.814 to 0.950
Black	0.893	0.015	0.815 to 0.979
Chinese	0.749	0.001	0.629 to 0.893
Other	0.817	<0.001	0.736 to 0.908

**Charlson Comorbidity Index score**			
0	1.000		
1	1.009	0.542	0.981 to 1.038
≥2	1.022	0.083	0.997 to 1.048

**IMD quintile**			
1 (least deprivation)	1.000		
2	1.020	0.260	0.985 to 1.056
3	1.014	0.415	0.980 to 1.050
4	1.017	0.332	0.983 to 1.052
5 (most deprivation)	1.033	0.044	1.001 to 1.067

*CXR = chest X-ray. HR = hazard ratio. IMD = Index of Multiple Deprivation.*

A similar, but weaker, association was seen when using the unadjusted CXR categories. The HR for patients in the ‘high’ group (≥40 CXRs/1000 patients) compared with the ‘low’ group (≤30 CXRs/1000 patients) was 0.98 (95% CI = 0.96 to 0.99, *P* = 0.004) (see Supplementary Table S4), but this was not maintained at 5 years when those who did not survive to 1 year were excluded (HR 0.99, 95% CI = 0.96 to 1.01, *P* = 0.338) (see Supplementary Table S5). Supplementary Table S6 details the results of the Cox analysis for 5-year survival with respect to unadjusted CXR, including those who did not survive to at least 1 year.

Participants in the age categories 60–64 years and younger had better 5-year survival, along with female patients and those in the Asian, Black, Chinese, and Other ethnic categories (Table 4). IMD quintiles 2 and 3 had favourable survival compared with IMD quintile 1 (least deprivation) at 1 year, with HRs of 0.96 (95% CI = 0.94 to 9.98, *P*<0.001) and 0.97 (95% CI = 0.95 to 0.99, *P* = 0.002), respectively (Table 3); at 5 years, however, this association was not evident (Table 4).

### Sensitivity and exploratory analyses

The results of the sensitivity analyses and exploratory analyses are presented in Supplementary Tables S7–S48 and Supplementary Figures S3 and S4. The sensitivity analyses yielded results that were consistent with those reported in the primary analyses. With respect to exploratory analyses, no evidence was found for a seasonal effect for cancer stage at diagnosis that was attributable to variations in CXR rate and emergency diagnosis through the year. This could be due to uneven fluctuations of CXR volumes, even if there were increases overall during winter, and because of varying duration from the point at which possible lung cancer was identified on the CXR to the date of diagnosis.

Evidence was found that patients who had higher numbers of CXRs in the 5 years prior to diagnosis, excluding the first year, were more likely to be diagnosed with early-stage disease: patients in the highest third of the CXR rates in that period had an OR of 0.82 with respect to late-stage diagnosis (95% CI = 0.76 to 0.87, *P*<0.001) (see Supplementary Table S41).

In order to explore the possibility of overdiagnosis, an exploratory analysis was undertaken (see Supplementary Table S48) to understand whether more cancers were found in practices with higher CXR use. Although this did not suggest overdiagnosis, that possibility cannot be entirely dismissed.

## Discussion

### Summary

Patients diagnosed with lung cancer who were registered at English general practices that had the highest utilisation of CXR were more likely to be diagnosed with early-stage disease and had improved 1-year and 5-year survival, compared with those registered at practices with the lowest utilisation of CXR. Female participants had favourable results regarding cancer stage at diagnosis and survival; participants in ethnic categories other than ‘Mixed’ had favourable survival compared with those in the White ethnic category. No consistent associations were observed with respect to deprivation.

### Strengths and limitations

This was a large study of >160 000 patients diagnosed with lung cancer, utilising a centrally administered and comprehensive national cancer registry. Accordingly, the authors were able to report meaningful outcome measures, namely 1-year and 5-year survival and cancer stage at diagnosis. Linkage of practice CXR rates was incorporated, using readily accessible routinely collected data in a novel manner. This is the first such study, to the authors’ knowledge, that has examined the relationship between the usual radiological investigation and the outcomes for the leading cause of cancer death. Analyses were undertaken in accordance with a detailed preregistered analysis plan.

Although available covariables were incorporated into logistic and Cox regression models at both practice and individual level, the possibility of confounding cannot be excluded. In particular, smoking status was not available for individuals. For practices, ethnicity and deprivation data were not derived directly from practice populations, but estimated from census and IMD data according to the geographical location of the practices.^16^ It is possible that practices that requested more CXRs were also more assiduous in other aspects of clinical care, and it is plausible that such practices also undertake more diagnostic testing — for example, blood testing and CT — in general.

Survival was defined as the time to event (death) from the date of diagnosis. The possibility that lead-time bias influenced the findings cannot be excluded. Under such a scenario, the chances of earlier diagnosis in practices that used more CXRs could have brought diagnoses forward, but without actually altering patients’ longevity. That earlier-stage disease (which is known to be much more amenable to curative treatment) was associated with increased CXR utilisation, the understanding of the relatively short preclinical sojourn time of lung cancer,^19^ and its tendency to progress rapidly compared with other cancers,^20^ suggests that the results do reflect clinically meaningful findings. Although an exploratory analysis did not demonstrate evidence for overdiagnosis, given the observational nature of this work, the possibility that overdiagnosis could have occurred and affected the results cannot be dismissed entirely.

For the unadjusted analyses, only three categories were used in order to enable a simple way to compare practices without the need for statistical adjustment. The thresholds for these categories were set close to each other (<30 CXRs/1000 patients, 30–39 CXRs/1000 patients, and ≥40 CXRs/1000 patients), which meant that improved stage at diagnosis and 1-year survival were found only for the highest unadjusted category and that no benefit was found for 5-year survival (when including only those who survived at least to 1 year).

### Comparison with existing literature

A large screening trial^9^ conclusively demonstrated that using CXR to test asymptomatic patients deemed to be at risk of lung cancer due to smoking history does not improve outcomes, so the NHS does not offer patients CXR without a specific indication (commonly, this constitutes symptoms suggestive of respiratory disease). As such, the population of this study are not equivalent to asymptomatic participants in screening trials and may have been at greater risk of lung cancer, given their symptoms and decision to consult their GP.

This study provides the most persuasive evidence of beneficial lung cancer outcomes associated with increased utilisation of CXR in symptomatic primary care populations to date. Three previous studies have considered whether such an effect might exist, arriving at conflicting conclusions, as outlined below.^21^^–^^23^

Cheyne *et al*^23^ obtained data on 1394 patients diagnosed with lung cancer in a single city in 2008–2010 and grouped patients into quintiles according to CXR rates of their general practices. No association was found between cancer stage at diagnosis or 1-year survival, although the study was insufficiently powered to detect modest differences.

O’Dowd *et al*^21^ obtained primary care data on 20 142 patients who were diagnosed with lung cancer between 2000 and 2013. Practices were ranked into quartiles according to standardised CXR rates, and analysis was undertaken with respect to the odds of death occurring within 90 days of diagnosis. Patients from practices in the highest quartile had an OR of 1.41 (95% CI = 1.29 to 1.55, *P*<0.001) of death within 90 days compared with practices in the lowest CXR rate quartile. This analysis did not incorporate linkage to cancer registry data, relying instead on this information being present in the primary care record.

Kennedy *et al*^22^ reported on a symptom awareness campaign in a large city, during which the annual volume of CXRs organised by general practices approximately doubled between 2008 and 2014. During this time, a 9% increase in the proportion of early-stage (stage I or II) lung cancer cases was observed, along with a 9% reduction in the absolute number of patients diagnosed with late-stage (stage III or IV) disease. Increases in 1-year survival were also observed, rising from 32% for 2008–2010 to 40% for 2013–2015. These improvements in outcome exceeded those seen nationally, but a temporal improvement independent of increasing CXR rates could not be excluded; in addition, the study reported on outcomes throughout the city, and not by rate of practice CXR utilisation.

The finding that patients with higher numbers of CXRs in the 5 years prior to diagnosis (excluding the first year) were more likely to be diagnosed with early-stage disease supports previous evidence from a cohort from a single centre, which suggested that individuals who were investigated more frequently with CXR — even in the period before a lung cancer was likely to have arisen — were more likely to be diagnosed with early-stage disease (see Supplementary Tables S41–S46).^24^

Several studies have examined variations in diagnostic activity in primary care, including for cancer.^12^^,^^25^^,^^26^ A much smaller number have examined whether variations in such activity between primary care organisations are associated with differences in cancer outcomes.^11^^,^^13^^,^^27^ The present study was preceded by a similar retrospective observation study of 22 488 cancer cases from 6513 general practices, which reported outcomes for oesophagogastric cancers using three categories of gastroscopy utilisation; patients in the lowest practice gastroscopy category were found to have higher rates of emergency admission and all-cause mortality at 1 year.^27^ The study did not report cancer stage or 5-year survival.

Finally, two cohort studies ^11^^,^^13^ examined general practice’s rates of referral for suspected cancer, rather than the investigations that typically underpin referral. The earlier of the two^11^ examined mortality of patients diagnosed with cancer, including 19 936 people who had lung cancer, according to general practice rate of urgent suspected cancer (USC) referral. Patients with lung cancer at practices in the lowest third of USC referral had an HR of 1.05 (95% CI = 1.02 to 1.08) relative to the intermediate group.^11^ The subsequent related study of cancer registrations between 2011 and 2015, which comprised 186 018 patients with lung cancer, found that the highest referring practices had favourable all-cause 5-year survival, including for lung cancer (HR 0.95, 95% CI = 0.94 to 0.97) when compared with the lowest referring practices and odds of stage I or II cancer at diagnosis (OR 0.92, 95% CI = 0.89 to 0.96).^13^ Neither cohort study addressed variation in CXR utilisation. As CXR is recommended for investigating almost all presentations of suspected lung cancer according to several guidelines worldwide,^7^ the propensity to request CXR is likely to be a critical factor driving the variation in lung cancer referrals, along with the impacts on cancer stage at diagnosis and survival observed in these cohort studies.

### Implications for practice

This study provides persuasive evidence that increased utilisation of CXR in primary care for patients who are symptomatic is associated with earlier-stage cancer at diagnosis and improved survival. The results resonate with previous research on testing with gastroscopy for oesophagogastric cancers and for practice referrals for USC; however, the findings of the present study, arguably, have much more immediate relevance for clinicians in primary care and cancer policy generally. Gastroscopy is relatively costly, and is invasive and burdensome for patients. USC referrals require clinic appointments and usually additional diagnostic tests, so general practices need to steward this resource carefully. In contrast, CXR is a relatively cheap non-invasive test, and is quick to perform and interpret.^28^ CXR utilisation can, therefore, be rapidly increased in ways not feasible for either gastroscopy or USC referrals. Indeed, experience has demonstrated that dramatic increases in the utilisation of CXR in primary care is readily achievable, when GPs or their patients are encouraged to lower their thresholds for investigation.^22^

Difficulties in accessing appointments in primary care may constitute an obstacle; however, mitigations exist, including open-access schemes.^29^^,^^30^ To obtain benefits, substantial additional CXRs would need to be undertaken, so policy would need to be tailored appropriately — for example, to support practices that are known to utilise CXRs infrequently and promote the test for those who have appropriate symptoms and risk factors, such as age and smoking status.

Previous research has demonstrated that clinical guidelines regarding symptoms that warrant referral for suspected cancer are inconsistently implemented in primary care.^31^^,^^32^ It may be possible to improve lung cancer outcomes with policy and practice initiatives that improve adherence to those guidelines that recommend imaging due to symptoms. Achieving earlier diagnosis remains a focus of cancer policy. Lung cancer is the UK’s third most common non-cutaneous malignancy, accounting for 13% of new cancer diagnoses.^33^ With only 29% of new diagnoses currently diagnosed at stage I or II,^34^ and the English health services’ aspiration of 75% of all cancers to be diagnosed at stage I or II by 2028, improvement is crucial if that gap is to be narrowed.^35^

The NHS in England has recommended that GPs have ‘direct access’ to investigations such as CT.^36^ This approach is predicated on the higher sensitivity of CT, as CXR does not identify around 20% of lung cancers.^37^^,^^38^ However, CT is much more costly than CXR, takes much longer for radiologists to interpret,^28^ and, consequently, access to the investigation is limited, particularly in the UK. The UK has relatively less imaging capacity than other high-income countries^10^ and the most common symptoms of lung cancer, such as cough, confer a risk of only around 1% for lung cancer;^39^ as such, CT may not be deemed suitable for such presentations. Increasing utilisation of CXR can be achieved much more readily than that for CT and this study suggests there are benefits to promoting investigation of patients who are symptomatic. This could be pursued as one part of a multipronged strategy, alongside direct access to CT and screening of patients who are asymptomatic with low-dose CT, as recommended in several jurisdictions worldwide, including by the UK’s National Screening Committee.^2^

Achieving earlier diagnosis of lung cancer is appropriately considered an urgent priority in cancer policy. Due to the typical symptom profile of lung cancer, in which low-risk symptoms predominate, prompt diagnosis is challenging. As lung cancer screening is likely to only identify a minority of cancers, investigation of patients who are symptomatic remains vital. Despite the limitations of CXR, this study has demonstrated that higher utilisation of the test is associated with improved outcomes. Given the substantial variation that exists in the rates of investigation with CXR in primary care, initiatives to increase CXR utilisation may be warranted as part of a wider strategy that includes lung cancer screening using low-dose CT, public awareness of symptoms, and greater availability of more-definitive modalities, such as CT.

## Data

This work uses data that have been provided by patients and collected by the NHS as part of their care and support. The data are collated, maintained, and quality assured by the National Cancer Registration and Analysis Service, which is part of the Health and Social Care Information Centre (NHS Digital). Access to the data was facilitated by the UK Health Security Agency Office for Data Release. Lung cancer registry data were made available for analysis under the terms of a data-sharing agreement; this does not permit the sharing of patient data. The general practice chest X-ray (CXR) rate categorisation data are available at: https://osf.io/pm6yh.
